# ICTV virus taxonomy profile: *Birnaviridae*

**DOI:** 10.1099/jgv.0.001185

**Published:** 2018-11-28

**Authors:** Bernard Delmas, Houssam Attoui, Souvik Ghosh, Yashpal S. Malik, Egbert Mundt, Vikram N. Vakharia, ICTV Report Consortium

**Affiliations:** ^1^​ VIM, INRA, Université Paris-Saclay, 78350, Jouy-en-Josas, France; ^2^​ UMR1161 Virologie, ANSES, INRA, Ecole Nationale Vétérinaire d'Alfort, Université Paris-Est, Maisons-Alfort, France; ^3^​ Department of Biomedical Sciences, Ross University School of Veterinary Medicine, St. Kitts and Nevis; ^4^​ Indian Veterinary Research Institute, Izatnagar 243 122, Uttar Pradesh, India; ^5^​ Boehringer Ingelheim Veterinary Research Center, Bemeroder Str. 31 30559 Hannover, Germany; ^6^​ Department of Marine Biotechnology, University of Maryland, Baltimore County, 701, East Pratt Street, Baltimore, MD 21202, USA

**Keywords:** *Birnaviridae*, ICTV Report, taxonomy

## Abstract

*Birnaviridae* is a family of viruses with bi-segmented dsRNA genomes totalling about 6 kbp forming icosahedral, non-enveloped virions. The family includes four genera, members of three of which (*Aquabirnavirus*, *Avibirnavirus* and *Blosnavirus*) infect vertebrates (excluding mammals), whereas members of the fourth genus (*Entomobirnavirus*) infect insects. Each genus includes 1–3 species. Infectious pancreatic necrosis virus of salmonids and infectious bursal disease virus of poultry are two economically important birnaviruses. This is a summary of the International Committee on Taxonomy of Viruses (ICTV) Report on the taxonomy of *Birnaviridae*, which is available at www.ictv.global/report/birnaviridae.

## Abbreviation

RdRP, RNA-dependent RNA polymerase.

## Virion

**Fig. 1.**
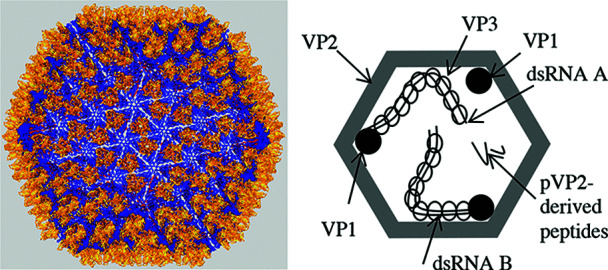
Structure of infectious bursal disease virus particles. (Left) three-dimensional model derived from X-ray crystallography (courtesy of F. Rey) with its T=13 *laevo* icosahedral shell. (Right) diagram of the distribution of polypeptides and virus genome in particles.

Virus particles are non-enveloped, single-shelled particles with a diameter of about 65 nm (Table 1, Fig. 1). The capsid follows a T=13 *laevo* icosahedral geometry and comprises a single capsid protein, VP2, clustered in trimers and forming 260 projections at the virus surface. A pseudo-atomic model of the virus particle at 3 Å resolution has been obtained by crystallography [1].

**Table 1.** Characteristics of the family *Birnaviridae*

Typical member: infectious bursal disease virus P2 (A: X84034; B: X84035), species *Infectious bursal disease virus*, genus *Avibirnavirus*	
Virion	Non-enveloped, icosahedral virion with internal ribonucleoprotein complexes
Genome	Two double-stranded RNA segments (2.9–3.6 kbp) with the RNA-dependent RNA-polymerase covalently linked to the 5′-end of the positive-sense strand
Replication	Cytoplasmic
Translation	Capped mRNAs, which lack poly(A) tracts, are translated by the cellular translation machinery
Host range	Vertebrates (excluding mammals); invertebrates
Taxonomy	Four genera: *Aquabirnavirus*, *Avibirnavirus*, *Blosnavirus* and *Entomobirnavirus*, each including one to three species

Inside the virus particle, ribonucleoprotein complexes made by the two genome segments associated with multiple copies of a ribonucleoprotein (VP3) and the RNA-dependent RNA polymerase (RdRP, VP1) do not follow the virus icosahedral symmetry [2].

## Genome

**Fig. 2.**
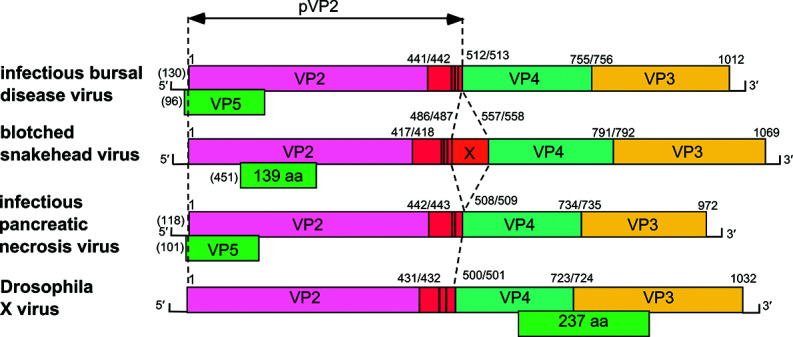
Schematic representation of the gene arrangement in the coding strand of genome segment A of birnaviruses, depicting representatives of the four genera: infectious bursal disease virus, infectious pancreatic necrosis virus, blotched snakehead virus and Drosophila X virus. Polyprotein cleavage sites are indicated by a vertical bar. The location and size of the small ORFs are indicated below the polyprotein ORF. Parentheses at the 5′-ends indicate the length of the 5′-non-coding region where known. Segment B of birnaviruses (not shown) encodes VP1 of 840 to 1050 aa.

The birnavirus genome is composed of two segments, with segment B coding for the viral RdRP, which is free in the virion or covalently attached to the genomic RNA segments by its N-terminal serine residue (as shown for infectious pancreatic necrosis virus) [3]. Segment A encodes the polyprotein precursor pVP2-VP4-VP3 and a separate small protein (VP5) (Fig. 2). VP4 is a protease that cleaves its own N- and C-termini in the polyprotein, thus releasing preVP2 and VP3 [4]. Subsequent serial cleavages at the C-terminus of preVP2 yield the mature VP2 protein and peptides that remain associated with the virion. For blotched snakehead virus and Tellina virus 1, an additional polypeptide (X) is located between the preVP2 and VP4 reading frames. The position of the VP5 ORF varies among birnaviruses.

## Replication

For binding of infectious bursal disease virus at the cell surface, proteins such as heat shock protein 90 and α4β2 integrin have been proposed to serve as receptors in chicken cells, with the virus amphiphilic peptide pep46 able to induce pores in target membranes. Endosomal acidification is not a prerequisite for virus internalization in infectious pancreatic necrosis virus-infected cells. The viral RdRP, which possesses a non-canonical active site [5, 6], becomes activated in the cytoplasm where two-genome-length mRNA molecules are produced from the dsRNA genome segments. These mRNAs are capped, and they lack 3′-poly(A) sequences. Virus assembly and pVP2 to VP2 maturation are concomitant and interdependent. In addition to VP4, virus assembly requires the recruitment of VP1 by VP3.

## Pathogenesis

The natural hosts of infectious pancreatic necrosis virus are salmonid fish and other freshwater and marine fishes. The virus is transmitted both vertically and horizontally. Its geographic distribution is worldwide; infections can result in high mortality in fry and fingerlings, which develop necrotic lesions in the pancreas. Infected adult fish become lifelong carriers without overt signs of infection.

The natural hosts of infectious bursal disease virus are chickens and turkeys. Infection affects the bursa of Fabricius in young chicks, causing B-lymphocyte depletion and subsequent severe immunosuppression. Genes involved in B-cell activation and signalling are downregulated following infection [7].

## Taxonomy

The genus *Entomobirnavirus* encompasses viruses isolated from insects, whereas members of the other genera have been isolated from non-mammalian vertebrates. Phylogenetic analyses of RdRP amino acid sequences reveal that birnaviruses are most closely related to members of the family *Permutotetraviridae* [5, 6]. Structural analysis of VP2 reveals homologies to the capsid proteins of members of the families *Nodaviridae* and *Tetraviridae*, confirming the phylogenetic link between positive-sense RNA viruses and birnaviruses [1]. The phylogeny of birnaviruses correlates well with their host at the phylum rank, but not at lower ranks [8].

## Resources

Full ICTV Report: www.ictv.global/report/birnaviridae.
